# Supplementary material for: FastqPuri: high-performance preprocessing of RNA-seq data
Source: BMC Bioinformatics. 2019 May 3;20:226. doi: 10.1186/s12859-019-2799-0 (PMC6500068; doi:10.1186/s12859-019-2799-0)
Supplement: Supplementary file 2 — Archive of FastqPuri. Archive containing all files needed to install and run FastqPuri v1.0.6. Date stamp March 22, 2019. (GZ 47,819 kb) [file 12859_2019_2799_MOESM2_ESM.gz › FastqPuri-1.0.6/html/struct__trimFilter_8c.html]

FastqPuri: src/struct\_trimFilter.c File Reference


FastqPuri


- src

Functions

struct\_trimFilter.c File Reference

function that frees the memory of parTF (structure storing the trimFilter/trimFilterDS input arguments).
More...

`#include "struct_trimFilter.h"`

Include dependency graph for struct\_trimFilter.c:

|  |  |
| --- | --- |
| Functions | |
| void | free\_parTF (Iparam\_trimFilter \*ptr\_parTF) |
|  | frees the allocated memory in Iparam\_trimFilter |
|  | |

## Detailed Description

function that frees the memory of parTF (structure storing the trimFilter/trimFilterDS input arguments).

Author
:   Paula Perez paula.nosp@m.pere.nosp@m.zrubi.nosp@m.o@gm.nosp@m.ail.c.nosp@m.om

Date
:   07.10.2017


---

Generated on Mon Mar 19 2018 23:42:01 for FastqPuri by  

 1.8.14
